# Nab-paclitaxel in combination with Bevacizumab in patients with non-squamous non-small cell lung cancer after failure of at least one prior systemic regimen

**DOI:** 10.7150/jca.47072

**Published:** 2020-09-13

**Authors:** Xuezhi Hao, Yixiang Zhu, Yuxin Mu, Shouzheng Wang, Junling Li, Puyuan Xing

**Affiliations:** 1 National Cancer Center/National Clinical Research Center For Cancer/Cancer Hospital, Chinese Academy of Medical Science and Peking Union Medical College, Beijing, 100021, China.; 2 Affiliated Hospital of Guizhou Medical University, Guizhou Province Tumor Hospital, Guiyang, P.R. China.

**Keywords:** carcinoma, non-small cell lung, nab-paclitaxel, bevacizumab, survival, adverse events

## Abstract

**Background:** Most patients with non-small cell lung cancer (NSCLC) experience disease progression after first-line treatment. The efficacy and safety of the nab-paclitaxel (nab-PTX) and bevacizumab combination as the second or further line of treatment in patients with advanced NSCLC have not been reported yet.

**Objective:** To evaluate the efficacy and safety of the nab-PTX and bevacizumab combination in patients with advanced non-squamous (NSQ) NSCLC after failure of at least one prior systemic regimen.

**Methods:** Patients with advanced (stage IV) NSQ NSCLC who received the nab-PTX and bevacizumab combination as the second or further line treatment between February 2012 and December 2018 at the Cancer Hospital of the Chinese Academy of Medical Sciences (Beijing, China) were included in this retrospective study. The main outcomes included the objective response rate (ORR), progression-free survival (PFS), overall survival (OS), and safety.

**Results:** Thirty-four patients received 1-27 cycles (median, four cycles) of treatment; 67.6% (23/34) patients had undergone at least two lines of previous treatment. The ORR and disease control rates were 26.5% (9/34) and 82.4% (28/34), respectively. The median PFS and OS were 6.0 (95% CI=2.9-7.2) and 11.0 (95% CI=7.8-18.7) months, respectively. The multivariable analyses indicated that the combined use of other drugs and pleural metastasis were respectively associated with better PFS (hazard ratio=0.354, 95% CI=0.134-0.935, *P=*0.036) and OS (hazard ratio=0.540, 95% CI=0.118-0.980, *P=*0.046). The most frequent grade 3-4 adverse events (AEs) were neutropenia 20.6% (7/34), leukopenia 8.8% (3/34), and anemia 5.9% (2/34). No grade 5 AE occurred.

**Conclusion:** Combined nab-PTX and bevacizumab might be an effective treatment regimen for patients with advanced NSQ NSCLC after failure of at least one prior systemic regimen, but studies have to validate those findings.

## Introduction

Non-small cell lung cancer (NSCLC) is the most common type of lung cancer 1, 2. NSCLC is often diagnosed at an advanced stage 3-5. Tyrosine kinase inhibitors (TKIs) targeting the epidermal growth factor receptor (EGFR) or anaplastic lymphoma kinase (ALK) have improved the overall survival (OS) of patients with the corresponding genetic alterations 6-9. Immune checkpoint inhibitors such as nivolumab or pembrolizumab as first-line treatments also significantly prolonged patient OS 10-12. Nevertheless, despite the efficacy of those drugs, treatment will eventually fail, the disease will progress 13, and the patients will need second and further lines of treatment 14, 15. The prognosis of patients with advanced NSCLC remains poor, especially for patients who received multiple lines of treatment 1, 2, 16.

Nab-paclitaxel (nab-PTX) is a nanoparticle formulation of paclitaxel bound to human serum albumin 17, 18. Compared with solvent-based paclitaxel (sb-PTX), nab-PTX can enhance drug delivery of the cytotoxic agent to tumors, increase the intra-tumor drug concentration 19, 20, and minimize the occurrence of hypersensitivity reactions due to the absence of the culprit solvent 21-23. The phase III open-label CA031 trial demonstrated that nab-PTX achieved a higher objective response rate (ORR) (33% vs. 25%) and a lower occurrence of grade 3-4 neutropenia (47% vs. 58%) compared with sb-PTX as first-line treatment for NSCLC 24. Based on the above results, the US FDA approved nab-PTX in combination with carboplatin for the first-line treatment of locally advanced or metastatic NSCLC. Real-world evidence also suggested that first-line treatment with nab-PTX plus carboplatin prolonged progression-free survival (PFS) and OS in patients with NSCLC 25, 26. In China, nab-PTX is mostly used as the second or further line treatment option because of limitations in indications, medical insurance, and costs, among others. A few studies reported that nab-PTX as a second or further line chemotherapy regimen was effective in advanced NSCLC in Western and East Asian populations 27-29. Nevertheless, there is no consensus or standardized protocol for the second or further line use of nab-PTX in patients with advanced NSCLC, and the exact effectiveness and safety data of nab-PTX in unknown.

Bevacizumab is a monoclonal antibody that inhibits angiogenesis by targeting the vascular epidermal growth factor (VEGF) 30. The addition of bevacizumab to carboplatin/paclitaxel (solvent-based chemotherapy) as a first-line regimen showed clinical benefits in the treatment of advanced NSCLC in several randomized controlled trials (RCTs) and has been recommended for patients without contraindications 31. The IFCT-1103 ULTIMATE trial showed that weekly sb-PTX with bevacizumab was superior to docetaxel as second- and third-line therapy for advanced NSCLC 32.

There is still a lack of evidence for the use of nab-PTX in combination with bevacizumab for the second or further line of treatment in patients with advanced NSCLC. Therefore, the aim of this study was to evaluate the efficacy and safety of the nab-PTX in combination with bevacizumab in patients with advanced non-squamous (NSQ) NSCLC after failure to at least one prior systemic regimen.

## Materials and Methods

### Study design and patients

This was a retrospective study of the patients with advanced (stage IV) NSCLC who received nab-PTX and bevacizumab as second or further line treatment between February 2012 and December 2018 at the Cancer Hospital of the Chinese Academy of Medical Sciences (CAMS) (Beijing, China). The study was approved by the Ethics Committee of the Cancer Hospital of the CAMS (Beijing, China) (approval number: 15-144/1071).

The inclusion criteria were: 1) received at least one line of systemic therapy for stage IV/metastatic NSCLC (either chemotherapy or TKIs); 2) received nab-PTX and bevacizumab in later-line regimen, for at least one cycle with radiological evaluation; 3) ≥18 years of age; 4) Eastern Cooperative Oncology Group (ECOG) performance status (PS) of 0-2; 5) pathologically confirmed non-squamous NSCLC; and 6) at least one measurable lesion according to RECIST 1.1. Patients who received a prior taxane were eligible except for nab-paclitaxel. The exclusion criterion was any contraindication to nab-paclitaxel or bevacizumab.

### Treatment

The 130-nm albumin-bound formulation of paclitaxel (nab-PTX [Abraxane]; Celgene, Summit, NJ, USA; 100 mg/vial) was routinely given at 130 mg/m^2^ over 30 min on days 1 and 8 while bevacizumab was given at a dose of 5-10 mg/kg on day 1 of a 21-day cycle. The patients were scheduled to receive at least two cycles, and the therapeutic efficacy was evaluated every two cycles. Patients with symptom aggravation after one cycle were also evaluated. Treatment was discontinued if progressive disease (PD) or intolerable adverse events (AEs) occurred.

### Data collection

The baseline patient data included age, sex, Eastern Cooperative Oncology Group (ECOG) performance status (PS), smoking history, stage, pathological type, number of previous treatment lines, number of treatment cycles of nab-PTX, prior taxane treatment, EGFR/ALK-mutation status, prior EGFR/ALK TKI treatment, and lung radiotherapy. PS was defined according to the ECOG performance scale 33. All tumor staging procedures were carried out using the 7th Union for International Cancer Control tumor node metastasis (TNM) classification.

### Treatment response and AEs

Tumor response was assessed by computed tomography (CT) scan, magnetic resonance imaging (MRI), bone scan, and tumor markers after every two treatment cycles. The Response Evaluation Criteria in Solid Tumors (RECIST) 1.1 criteria were used for treatment response evaluation in terms of complete response (CR), partial response (PR), stable disease (SD), PD, ORR (as CR+PR), and disease control rate (DCR) (as CR+PR+SD). Any AE that occurred between the initiation of treatment and until 1 month after ending treatment was recorded as an AE, regardless of whether the AE was associated with the drug. The evaluation of AEs was based on the National Cancer Institute-Common Toxicity Criteria (NCI-CTC) 3.0 version.

### Outcomes

The main outcomes included the ORR, PFS, OS, and AEs. These data were obtained from multiple sources, including clinical letters, follow-up examinations, and hospital computer information systems. PFS was determined as the time from the start of treatment to disease progression (local, regional, and/or distant) or death from any cause, whichever occurred first. OS was defined as the time from the start of treatment to death from any cause.

### Statistical analysis

The patients' characteristics and treatment responses were analyzed using descriptive statistics. Continuous variables are presented as means ± standard deviation and were analyzed using the Student t-test. Categorical variables are presented as frequencies and were analyzed using the chi-square test. PFS and OS were calculated with the Kaplan-Meier method. The frequencies of AEs were summarized using absolute frequencies and percentages. The Cox proportional hazard regression model was used to identify the risk factors associated with disease progression. In univariable analyses, all baseline variables were examined for association. In the multivariable analyses, the factors with *P*-values <0.20 in the univariable analyses were included. All statistical analyses were performed using SPSS 17 (IBM, Armonk, NY, USA). *P* values <0.05 were considered statistically significant.

## Results

### Characteristics of the patients

From February 2012 to December 2018, 34 patients received 1-27 cycles (median, four cycles) of treatment. Their characteristics are listed in **Table 1.** Among them, 28 (82.4%) patients were <65 years of age; five (14.7%) were ECOG PS 0 and 29 (85.3%) were PS 1; 21 (61.8%) were male; and 15 (44.1%) had a history of smoking. Most patients (n=32; 94.1%) had adenocarcinoma; 12 (35.3%) patients had confirmed EGFR mutation, while two (5.9%) had ALK translocation. The brain and pleural metastasis rates were 26.5% (n=9) and 50.0% (n=17), respectively. Regarding previous treatments, 16 (47.1%) patients were treated with a taxane and 16 (47.1%) with an EGFR/ALK TKI; 23 (67.6%) patients had received one or two lines of chemotherapy prior to nab-PTX/bevacizumab, and 23 (67.6%) patients had received radiotherapy. Twelve patients received other drugs in addition to nab-PTX and bevacizumab: one (2.9%) received sunitinib, two (5.9%) received gemcitabine, two (5.9%) received immune checkpoint inhibitors, and seven (20.6%) received platinum. The median follow-up for all patients was 6.8 months without censoring or was not reached when using the reverse Kaplan-Meier method.

### Tumor response and survival

As shown in **Table 2**, the ORR and DCR were 26.5% (9/34) and 82.4% (28/34), respectively. CR was not observed, while the PR rate was 26.5% (9/34). The SD and PD rates were 55.9% (19/34) and 17.6% (6/34), respectively.

**Figure 1:**
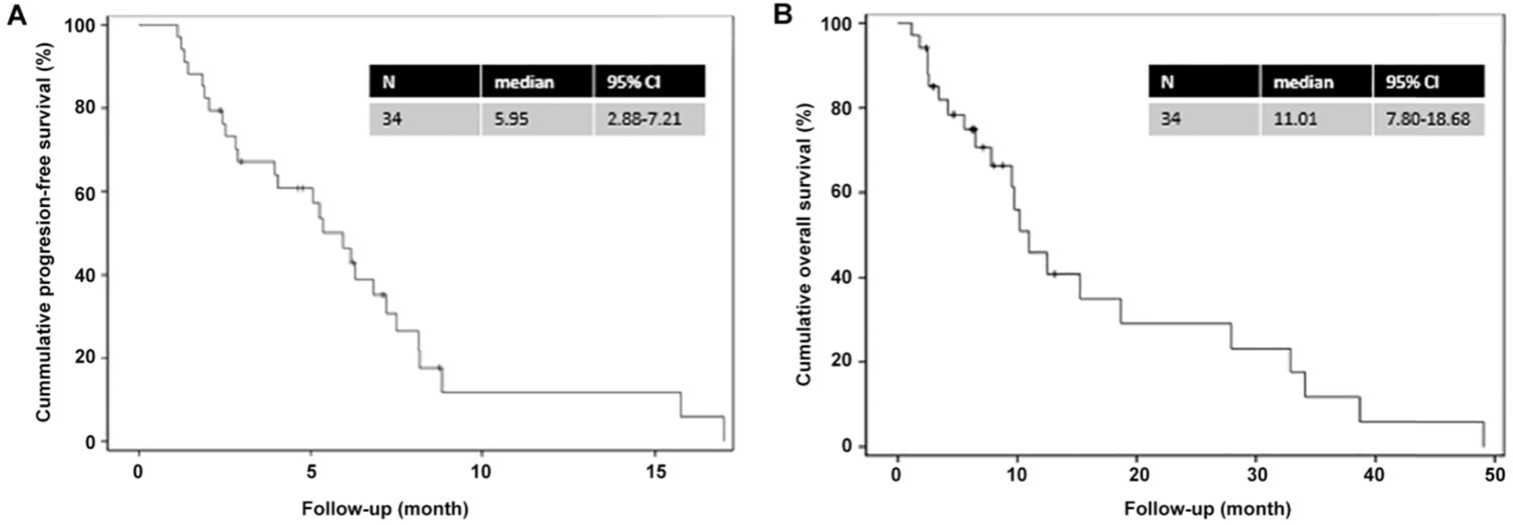
Kaplan-Meier curves for progression-free survival and overall survival. (A) Progression-free survival; (B) Overall survival.

The Kaplan-Meier analysis (**Figure 1**) showed that the median PFS and OS were 6.0 (95% CI=2.9-7.2) and 11.0 (95% CI=7.8-18.7) months, respectively. The 1-year PFS rate and 1-year OS rates were 10% and 40%, respectively, while the 2-year OS rate was 30%.

### Factors associated with PFS and OS

As shown in **Table 3**, in the univariable analyses for PFS and OS, only pleural metastasis (*P=*0.029) was significantly associated with OS. In the multivariable analyses, the combined use of other drugs was associated with better PFS (HR=0.354, 95%CI=0.134-0.935, *P=*0.036), and pleural metastasis was associated with better OS (HR=0.540, 95%CI=0.118-0.980, *P=*0.046) (**Table 4**).

### AEs

Regarding hematological AEs, the majority were of mild severity. The occurrence rates of grade 3-4 of anemia, neutropenia, leukopenia, and peripheral were 5.9%, 20.5%, 8.8%, and 2.9%, respectively. No other grade 3-4 hematologic or non-hematological AEs were observed. No grade 5 AE occurred in the present study.

## Discussion

The efficacy and safety of nab-PTX and bevacizumab as a second or further line of treatment in patients with advanced NSCLC have not been reported yet. Therefore, this study aimed to evaluate the efficacy and safety of nab-PTX in combination with bevacizumab in patients with advanced NSQ NSCLC after failure of at least one prior systemic regimen. The results strongly suggest that the combined nab-PTX and bevacizumab might be an effective treatment regimen for patients with advanced NSQ NSCLC after failure of at least one prior systemic regimen, but studies have to validate those findings. In addition, the toxicity profile was, in general, mild and manageable.

Currently, there is no standardized regimen for the use of nab-PTX as a second or further line of treatment for NSCLC. A total dose of 260-300 mg/m^2^ of nab-PTX is usually administered for one treatment cycle of 3 weeks, with or without other anti-tumor drugs. Bevacizumab is a recognized add-on therapy for NSCLC 15, 34-36. To our knowledge, this is the first study that evaluated the efficacy and safety of nab-PTX in combination with bevacizumab for the treatment of patients with NSQ NSCLC after failure to at least one prior systemic regimen. The results showed that the ORR and DCR were 26.5% and 82.4%, respectively; the median PFS and OS were 6.0 and 11.0 months, respectively. Previous studies of nab-PTX alone in second or further line treatment of NSCLC reported a PFS of 3.5-6.6 months and an OS of 6.8-15.7 months, with ORRs ranging from 16.1%-35.5% 27, 28, 37-40. A study of nab-PTX combined with carboplatin reported a PFS of 4.0 months and an OS of 14.0 months 41. Finally, a study of nab-PTX combined with pemetrexed showed a PFS of 4.4 months, an OS of 8.8 months, and an ORR of 14% 42. Three studies of PTX combined with bevacizumab in previously treated patients with NSCLC reported ORRs of 40.0%-48.8%, DCRs of 75.0%-86.0%, a PFS of 4.6-6.4 months, and an OS of 9.6-14.5 months 43-45. The treatment effects observed in the present study for the nab-PTX and bevacizumab combination were generally consistent with these previous results. The good effects of nab-PTX with bevacizumab might be due to the complementary mechanisms of the two drugs. Indeed, paclitaxel is an agent disrupting the normal functioning of the microtubules, preventing cell division and leading to apoptosis; it also has anti-angiogenic effects, probably due to the inhibition of the proliferation of endothelial cells. Bevacizumab is an anti-angiogenesis agent that directly blocks the action of VEGFR 43-45. Animal studies showed that VEGF could reverse the effects of docetaxel, another member of the taxane family along with PTX and that the use of an anti-VEGF drug could restore the activity of docetaxel 46. Furthermore, Shaked et al. 47 showed that PTX could induce circulating endothelial progenitor mobilization and promote angiogenesis, whereas the combined use of anti-angiogenic drugs such as bevacizumab could enhance the efficacy of PTX. Combined together, the two drugs probably have a profound synergistic effect on the tumor's blood vessels 45. It has also been suggested that bevacizumab could normalize the tumor blood vessels, improving drug delivery and the immune microenvironment. In addition, oxygenation could be improved, preventing tumor hypoxia and hypoxia-inducible factor-mediated chemotherapy and radiotherapy resistance 48-51. Nevertheless, beyond the clinical benefits that can be observed with the combination, the exact mechanisms for the synergy between bevacizumab and PTX on NSCLC and other solid tumors, in general, remain to be determined.

With regard to factors associated with patient prognosis, unexpectedly, a positive association between the presence of pleural metastasis and better OS was found. This association might be due to an improved response to the nab-PTX plus bevacizumab regimen in patients with pleural metastasis. Bevacizumab injection into the pleural cavity has been used for the management of pleural effusion and showed good benefits 52. The combination of sb-PTX with bevacizumab has also been shown to be better than docetaxel for the management of pleural effusion from NSCLC 53. As an anti-angiogenesis agent, bevacizumab could, at least theoretically, decrease fluid accumulation in the pleural cavity, but there are some controversies regarding this effect 54. Alternatively, the positive association between pleural metastasis and better OS could be a result of the small sample size and sampling bias. The concomitant use of other drugs was another independent factor identified to be associated with improving survival. In the present study, all patients received nab-PTX in combination with bevacizumab, but some patients also received short courses of other concurrent anti-tumor drugs (sunitinib (n=1), gemcitabine (n=2), immune checkpoint inhibitor (n=2), or platinum (n=7)), according to the treating physician's discretion and the patient's condition. Combining multiple drugs may offer the opportunity to fight the cancer cells at multiple frontlines at once, and therefore circumvent possible resistance, but at the probable cost of increased toxicity. Further trials are needed to validate the effects of multiple drug combinations.

In the present study, the main grade 3-4 AEs were neutropenia (20.6%), leukopenia (8.8%), and anemia (5.9%), which can be considered as a good AE profile. This is also consistent with the known safety profiles of nab-PTX and bevacizumab 21-23, 30. Using nab-PTX alone, the rates of grade 3-4 leukopenia, neutropenia, and anemia have been reported to be 5%-49%, 2%-69%, and 0%-32% 24, 27, 28, 37-40. In a study of nab-PTX combined with carboplatin, neutropenia, thrombocytopenia, and anemia were observed in 28.0%, 12.0%, and 8.0% of the patients, respectively 41. A study of nab-PTX combined with pemetrexed showed 8.2% of anemia, 6.1% of leukopenia, and 10.2% of neutropenia 42. In two studies of sb-PTX with bevacizumab, the rates of neutropenia were 50.0% and 19.3% 53. In PTX combined with bevacizumab, the rates of neutropenia were 20%-37.5% 43-45. The toxicity profile in the present study falls within the reported ranges but in the lower end of those ranges. Nevertheless, as a retrospective study, an underreporting bias is always possible as some grade 3-4 AEs might have been treated at other hospitals.

The present study has limitations. The sample size was relatively small, and 41% of the patients had *EGFR/ALK* mutations. The patients received a wide variety of previous treatment regimens and concurrent supportive therapies, preventing further subgroup analyses and introducing bias. Bevacizumab was administered at two dose levels, as per physicians' choice and experience. Importantly, retrospective studies carry a risk of underreporting of the AEs. Such studies are limited to the data that can be found in the charts. Retrospective studies provide limited conclusions, but they can nevertheless provide useful insights for future clinical trials. The results should be confirmed by a multicenter study.

## Conclusion

In conclusion, combined nab-PTX and bevacizumab might be an effective treatment regimen for patients with advanced NSQ NSCLC after failure of at least one prior systemic regimen, but studies have to validate those findings.

## Figures and Tables

**Table 1:** Characteristics of the patients (n=34)

	n	%
**Age**		
≤65	28	82.4
>65	6	17.6
**ECOG PS**		
0	5	14.7
1	29	85.3
**Sex**		
Female	13	38.2
Male	21	61.8
**Smoker**		
Yes	15	44.1
No	18	52.9
Unknown	1	2.9
**Tumor pathological type**		
Adenocarcinoma	32	94.1
Adenosquamous carcinoma	2	5.9
**EGFR mutation**		
Mutant	12	35.3
Wild-type	18	52.9
Unknown	4	11.8
**ALK translocation**		
Yes	2	5.9
No	17	50.0
Unknown	15	44.1
**Brain metastasis**		
Yes	9	26.5
No	25	73.5
**Pleural metastasis**		
Yes	17	50.0
No	17	50.0
**Previous lines of systemic therapy**		
1-2	23	67.6
≥3	11	32.4
**Previous taxane**		
Yes	16	47.1
No	18	52.9
**Previous EGFR/ALK TKI**		
Yes	16	47.1
No	18	52.9
**Previous lines of chemotherapy**		
1-2	23	67.6
≥3	11	32.4
**Previous radiotherapy**		
Yes	11	32.4
No	23	67.6

ECOG PS: Eastern Cooperative Oncology Group performance status; EGFR: epidermal growth factor receptor; ALK: anaplastic lymphoma kinase; TKI, tyrosine kinase inhibitors.

**Table 2:** Best overall response (n=34)

	n	%
**Best overall response**		
Complete response	0	0
Partial response	9	26.5
Stable disease	19	55.9
Progressive disease	6	17.6
**Overall response**	9	26.5
**Disease control**	28	82.4

**Table 3:** Univariable analysis of factors associated with progression-free survival and overall survival

Variables	HR	95%CI	*P*
**PFS**			
Age (≤65 years vs. >65 years)	1.274	0.288-5.626	0.749
Sex (male vs. female)	0.773	0.348-1.713	0.525
Smoking (smoker vs. non-smoker)	0.696	0.306-1.582	0.387
ECOG PS (1 vs. 0)	2.395	0.687-8.349	0.171
Previous ALK/EGFR-TKI (yes vs. no)	1.268	0.573-2.805	0.558
Brain metastasis (yes vs. no)	2.287	0.955-5.477	0.063
Pleural metastasis (yes vs. no)	0.682	0.312-1.489	0.337
EGFR/ALK mutation (yes vs. no)	1.278	0.578-2.826	0.545
Lines of previous chemotherapy (1-2 vs. ≥3)	0.516	0.221-1.207	0.127
Previous taxane (yes vs. no)	1.572	0.718-3.444	0.258
Previous radiotherapy (yes vs. no)	1.334	0.567-3.135	0.509
Combination with other drugs (yes vs. no)	0.517	0.214-1.250	0.143
**OS**			
Age (≤65 years vs. >65 years)	1.348	0.168-10.796	0.779
Sex (male vs. female)	1.928	0.786-4.733	0.152
Smoking (smoker vs. non-smoker)	1.417	0.596-3.369	0.431
ECOG PS (1 vs. 0)	1.635	0.533-5.013	0.390
Previous ALK/EGFR-TKI (yes vs. no)	0.722	0.302-1.727	0.464
Brain metastasis (yes vs. no)	1.121	0.400-3.145	0.828
Pleural metastasis (yes vs. no)	0.311	0.109-0.887	0.029
EGFR/ALK mutation (yes vs. no)	1.040	0.435-2.486	0.929
Lines of previous chemotherapy (1-2 vs. ≥3)	0.625	0.224-1.747	0.370
Previous taxane (yes vs. no)	0.886	0.368-2.133	0.787
Previous radiotherapy (yes vs. no)	2.300	0.773-6.843	0.135
Combination with other drugs (yes vs. no)	0.716	0.276-1.858	0.493

Data were analyzed using Cox regression. PFS: progression-free survival; OS: overall survival; HR: hazard ratio; CI: confidence interval; ECOG PS: Eastern Cooperative Oncology Group performance status; EGFR: epidermal growth factor receptor; ALK: anaplastic lymphoma kinase; TKI: tyrosine kinase inhibitors.

**Table 4:** Multivariable analysis of factors associated with progression-free survival and overall survival

Variables	HR	95% CI	*P*
PFS			
Brain metastasis (yes vs. no)	1.638	0.525-5.111	0.396
Lines of previous chemotherapy (1-2 vs. ≥3)	0.495	0.160-1.527	0.221
Combination with other drugs (yes vs. no)	0.354	0.134-0.935	0.036
OS			
ECOG PS (1 vs. 0)	2.501	0.660-9.475	0.177
Sex (male vs. female)	1.426	0.540-3.768	0.474
Pleural metastasis (yes vs. no)	0.540	0.118-0.980	0.046
Previous radiotherapy (yes vs. no)	3.768	0.573-6.072	0.300

Covariates with *P* values <0.20 in univariable Cox proportional hazard model were included in the multivariable Cox proportional hazard model. PFS: progression-free survival; OS: overall survival; HR: hazard ratio; CI: confidence interval; ECOG PS: Eastern Cooperative Oncology Group performance status; EGFR: epidermal growth factor receptor; ALK: anaplastic lymphoma kinase.

**Table 5:** Adverse events in all patients (n=34)

	Any grade	Grade 1	Grade2	Grade 3	Grade 4
**Hematologic adverse events**					
Anemia	12 (35.3)	8 (23.5)	2 (5.9)	2 (5.9)	0
Neutropenia	15 (44.1)	7 (20.6)	1 (2.9)	6 (17.6)	1 (2.9)
Leukopenia	17 (50.0)	8 (23.5)	6 (17.6)	3 (8.8)	0
Thrombocytopenia	2 (5.9)	2 (5.9)	0	0	0
Hemorrhinia	2 (5.9)	2 (5.9)	0	0	0
High blood pressure	0	0	0	0	0
**Non-hematologic adverse events**					
Peripheral neurotoxicity	6 (17.6)	3 (8.8)	2 (5.9)	1 (2.9)	0
Vomiting	17 (50.0)	11 (32.4)	6 (17.6)	0	0
Lipsotrichia	4 (11.8)	3 (8.8)	1 (2.9)	0	0
Myalgia	2 (5.9)	2 (5.9)	0	0	0
Fatigue	3 (8.8)	1 (2.9)	2 (5.9)	0	0
Increased ALT/AST	5 (14.7)	5 (14.7)	0	0	0
Fever	0	0	0	0	0
Mucositis	0	0	0	0	0
Diarrhea	0	0	0	0	0
Constipation	0	0	0	0	0

All data are shown as n (%). ALT, alanine transaminase; AST, aspartate aminotransferase.

## Abbreviations

NSCLC: non-small cell lung cancer
nab-PTX: nab-paclitaxel
NSQ: non-squamous
ORR: objective response rate
PFS: progression-free survival
OS: overall survival
AEs: adverse events
TKIs: Tyrosine kinase inhibitors
EGFR: epidermal growth factor receptor
ALK: anaplastic lymphoma kinase
sb-PTX: solvent-based paclitaxel
PFS: progression-free survival
VEGF: vascular epidermal growth factor
RCTs: randomized controlled trials
CAMS: Chinese Academy of Medical Sciences
PD: progressive disease
ECOG: Eastern Cooperative Oncology Group
PS: performance status
TNM: tumor node metastasis
RECIST: Response Evaluation Criteria in Solid Tumors
CR: complete response
PR: partial response
SD: stable disease
NCI-CTC: National Cancer Institute-Common Toxicity Criteria
